# 2-(2-Meth­oxy­phen­oxy)-3-nitro­pyridine

**DOI:** 10.1107/S1600536811045247

**Published:** 2011-11-05

**Authors:** Shah Bakhtiar Nasir, Zainal Abidin Fairuz, Zanariah Abdullah, Seik Weng Ng, Edward R. T. Tiekink

**Affiliations:** a Department of Chemistry, University of Malaya, 50603 Kuala Lumpur, Malaysia; b Chemistry Department, Faculty of Science, King Abdulaziz University, PO Box 80203 Jeddah, Saudi Arabia

## Abstract

In the title compound, C_12_H_10_N_2_O_4_, the pyridine and benzene rings are almost orthogonal, forming a dihedral angle of 86.63 (6)°. Each of the nitro [O—N—C—C torsion angle = −6.45 (19)°] and meth­oxy [C—O—C—C torsion angle = 179.69 (11)°] groups is almost coplanar with the ring to which it is connected. Mol­ecules are consolidated in the crystal structure *via* C—H⋯O inter­actions, forming a three-dimensional network.

## Related literature

For the structure of a related nitro-pyridine derivative, see: Nasir *et al.* (2010 ▶).
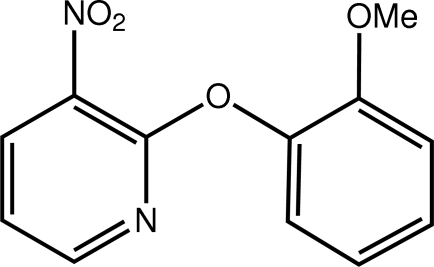


## Experimental

### 

#### Crystal data

C_12_H_10_N_2_O_4_
*M* *_r_* = 246.22
Monoclinic, 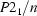

*a* = 7.5017 (7) Å
*b* = 7.1542 (6) Å
*c* = 20.6369 (18) Å
β = 91.878 (1)°
*V* = 1106.96 (17) Å^3^
*Z* = 4
Mo *K*α radiation
μ = 0.11 mm^−1^
*T* = 100 K
0.35 × 0.30 × 0.20 mm

#### Data collection

Bruker SMART APEX CCD diffractometer
Absorption correction: multi-scan (*SADABS*; Sheldrick, 1996 ▶) *T*_min_ = 0.961, *T*_max_ = 0.978
10026 measured reflections
2551 independent reflections
2103 reflections with *I* > 2σ(*I*)
*R*_int_ = 0.029

#### Refinement

*R*[*F* ^2^ > 2σ(*F* ^2^)] = 0.037
*wR*(*F* ^2^) = 0.103
*S* = 1.03
2551 reflections
164 parameters
H-atom parameters constrained
Δρ_max_ = 0.25 e Å^−3^
Δρ_min_ = −0.25 e Å^−3^

### 

Data collection: *APEX2* (Bruker, 2009 ▶); cell refinement: *SAINT* (Bruker, 2009 ▶); data reduction: *SAINT*; program(s) used to solve structure: *SHELXS97* (Sheldrick, 2008 ▶); program(s) used to refine structure: *SHELXL97* (Sheldrick, 2008 ▶); molecular graphics: *ORTEP-3* (Farrugia, 1997 ▶) and *DIAMOND* (Brandenburg, 2006 ▶); software used to prepare material for publication: *publCIF* (Westrip, 2010 ▶).

## Supplementary Material

Crystal structure: contains datablock(s) global, I. DOI: 10.1107/S1600536811045247/hb6471sup1.cif

Structure factors: contains datablock(s) I. DOI: 10.1107/S1600536811045247/hb6471Isup2.hkl

Supplementary material file. DOI: 10.1107/S1600536811045247/hb6471Isup3.cml

Additional supplementary materials:  crystallographic information; 3D view; checkCIF report

## Figures and Tables

**Table 1:** Hydrogen-bond geometry (Å, °)

*D*—H⋯*A*	*D*—H	H⋯*A*	*D*⋯*A*	*D*—H⋯*A*
C3—H3⋯O2^i^	0.95	2.58	3.4085 (17)	146
C9—H9⋯O4^ii^	0.95	2.55	3.2659 (16)	132
C12—H12a⋯O3^iii^	0.98	2.52	3.3560 (18)	143

Symmetry codes: (i) 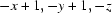; (ii) 
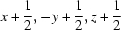
; (iii) 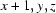.

**Fig. 1.**
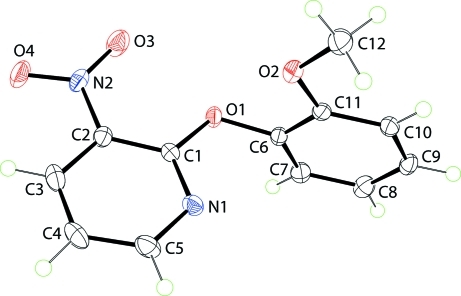
The molecular structure of (I) showing displacement ellipsoids at the 50% probability level.

**Fig. 2.**
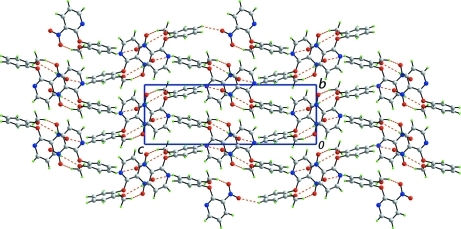
Unit-cell contents for (I) shown in projection down the a axis. The C—H···O interactions are shown as orange dashed lines.

## supplementary crystallographic information

### Comment

As a continuation of synthetic and structural studies of nitro-pyridine derivatives (Nasir *et al.*, 2010), the title compound, (I), was investigated, Fig. 1. The dihedral angle formed between the pyridine and benzene rings is 86.63 (6)°, indicating an almost orthogonal relationship. Each of the nitro [the O3—N2—C2—C1 torsion angle is -6.45 (19)°] and methoxy [the C12—O2—C11—C6 torsion angle is 179.69 (11)°] groups is co-planar with the ring to which it is attached. Molecules are stabilized in the three-dimensional crystal structure by C—H···O interactions, Table 1. Globally, the nitro-pyridine residues pack into layers in the *ab* plane with the benzene rings projecting to either side, Fig.2.

### Experimental

*o*-Methoxyphenol (2.5 g, 20 mmol) and sodium hydroxide (0.80 g, 20 mmol) were dissolved in water (50 ml) and to the solution was added 2-chloro-3-nitropyridine (3.17 g, 20 mmol) dissolved in THF (50 ml). The mixture was heated for 4 h. Water was added and the organic phase extracted with chloroform. The chloroform solution was dried over sodium sulfate and evaporation of the solvent yielded colourless blocks.

### Refinement

Hydrogen atoms were placed at calculated positions (C—H 0.95–0.98 Å) and were treated as riding on their parent carbon atoms, with *U*(H) set to 1.2–1.5 times *U*_eq_(C).

### Crystal data

**Table:**

C_12_H_10_N_2_O_4_	*F*(000) = 512
*M**_r_* = 246.22	*D*_x_ = 1.477 Mg m^−^^3^
Monoclinic, *P*2_1_/*n*	Mo *K*α radiation, λ = 0.71073 Å
Hall symbol: -P 2yn	Cell parameters from 3556 reflections
*a* = 7.5017 (7) Å	θ = 2.9–28.2°
*b* = 7.1542 (6) Å	µ = 0.11 mm^−^^1^
*c* = 20.6369 (18) Å	*T* = 100 K
β = 91.878 (1)°	Block, colourless
*V* = 1106.96 (17) Å^3^	0.35 × 0.30 × 0.20 mm
*Z* = 4	

### Data collection

**Table:**

Bruker SMART APEX CCD diffractometer	2551 independent reflections
Radiation source: fine-focus sealed tube	2103 reflections with *I* > 2σ(*I*)
graphite	*R*_int_ = 0.029
ω scans	θ_max_ = 27.5°, θ_min_ = 2.0°
Absorption correction: multi-scan (*SADABS*; Sheldrick, 1996)	*h* = −9→9
*T*_min_ = 0.961, *T*_max_ = 0.978	*k* = −9→9
10026 measured reflections	*l* = −23→26

### Refinement

**Table:**

Refinement on *F*^2^	Primary atom site location: structure-invariant direct methods
Least-squares matrix: full	Secondary atom site location: difference Fourier map
*R*[*F*^2^ > 2σ(*F*^2^)] = 0.037	Hydrogen site location: inferred from neighbouring sites
*wR*(*F*^2^) = 0.103	H-atom parameters constrained
*S* = 1.03	*w* = 1/[σ^2^(*F*_o_^2^) + (0.053*P*)^2^ + 0.3772*P*] where *P* = (*F*_o_^2^ + 2*F*_c_^2^)/3
2551 reflections	(Δ/σ)_max_ < 0.001
164 parameters	Δρ_max_ = 0.25 e Å^−^^3^
0 restraints	Δρ_min_ = −0.25 e Å^−^^3^

### Special details

**Table:**

Geometry. All s.u.'s (except the s.u. in the dihedral angle between two l.s. planes) are estimated using the full covariance matrix. The cell s.u.'s are taken into account individually in the estimation of s.u.'s in distances, angles and torsion angles; correlations between s.u.'s in cell parameters are only used when they are defined by crystal symmetry. An approximate (isotropic) treatment of cell s.u.'s is used for estimating s.u.'s involving l.s. planes.
Refinement. Refinement of *F*^2^ against ALL reflections. The weighted *R*-factor *wR* and goodness of fit *S* are based on *F*^2^, conventional *R*-factors *R* are based on *F*, with *F* set to zero for negative *F*^2^. The threshold expression of *F*^2^ > 2σ(*F*^2^) is used only for calculating *R*-factors(gt) *etc*. and is not relevant to the choice of reflections for refinement. *R*-factors based on *F*^2^ are statistically about twice as large as those based on *F*, and *R*- factors based on ALL data will be even larger.

### Fractional atomic coordinates and isotropic or equivalent isotropic displacement parameters (Å^2^)

**Table:**

	*x*	*y*	*z*	*U*_iso_*/*U*_eq_	
O1	0.36667 (12)	0.22735 (12)	0.12197 (4)	0.0182 (2)	
O2	0.71311 (12)	0.13613 (14)	0.12236 (4)	0.0206 (2)	
O3	0.20311 (15)	0.16998 (15)	0.01073 (5)	0.0316 (3)	
O4	0.12030 (15)	0.40621 (16)	−0.04768 (5)	0.0323 (3)	
N1	0.47657 (14)	0.52273 (15)	0.13850 (5)	0.0190 (2)	
N2	0.20013 (15)	0.33758 (17)	−0.00044 (5)	0.0207 (3)	
C1	0.38285 (16)	0.40572 (17)	0.10136 (6)	0.0150 (3)	
C2	0.29836 (16)	0.46433 (18)	0.04319 (6)	0.0171 (3)	
C3	0.30952 (18)	0.6508 (2)	0.02541 (7)	0.0235 (3)	
H3	0.2519	0.6939	−0.0135	0.028*	
C4	0.4052 (2)	0.7729 (2)	0.06477 (7)	0.0262 (3)	
H4	0.4140	0.9017	0.0541	0.031*	
C5	0.48791 (18)	0.70151 (19)	0.12028 (7)	0.0236 (3)	
H5	0.5568	0.7842	0.1470	0.028*	
C6	0.45474 (17)	0.18151 (17)	0.18111 (6)	0.0165 (3)	
C7	0.35938 (17)	0.17835 (18)	0.23680 (6)	0.0198 (3)	
H7	0.2382	0.2170	0.2360	0.024*	
C8	0.44250 (18)	0.11778 (19)	0.29441 (7)	0.0217 (3)	
H8	0.3787	0.1162	0.3334	0.026*	
C9	0.61791 (18)	0.06017 (18)	0.29447 (6)	0.0196 (3)	
H9	0.6736	0.0169	0.3337	0.024*	
C10	0.71438 (17)	0.06438 (17)	0.23835 (6)	0.0179 (3)	
H10	0.8354	0.0250	0.2392	0.022*	
C11	0.63311 (17)	0.12668 (17)	0.18063 (6)	0.0160 (3)	
C12	0.89664 (19)	0.0810 (2)	0.12152 (7)	0.0250 (3)	
H12A	0.9384	0.0882	0.0771	0.038*	
H12B	0.9088	−0.0477	0.1374	0.038*	
H12C	0.9683	0.1646	0.1495	0.038*	

### Atomic displacement parameters (Å^2^)

**Table:**

	*U*^11^	*U*^22^	*U*^33^	*U*^12^	*U*^13^	*U*^23^
O1	0.0237 (5)	0.0134 (4)	0.0169 (5)	−0.0025 (4)	−0.0074 (4)	0.0026 (3)
O2	0.0236 (5)	0.0230 (5)	0.0152 (5)	0.0025 (4)	−0.0015 (4)	0.0007 (4)
O3	0.0442 (6)	0.0264 (6)	0.0235 (6)	−0.0106 (5)	−0.0096 (5)	0.0015 (4)
O4	0.0353 (6)	0.0440 (7)	0.0170 (5)	0.0105 (5)	−0.0087 (4)	0.0004 (5)
N1	0.0201 (5)	0.0141 (5)	0.0226 (6)	−0.0003 (4)	−0.0027 (4)	−0.0002 (4)
N2	0.0199 (5)	0.0292 (7)	0.0129 (5)	0.0011 (5)	−0.0003 (4)	0.0006 (5)
C1	0.0153 (6)	0.0132 (6)	0.0165 (6)	0.0011 (4)	0.0009 (5)	0.0009 (5)
C2	0.0167 (6)	0.0196 (7)	0.0149 (6)	0.0013 (5)	0.0006 (5)	0.0009 (5)
C3	0.0247 (7)	0.0230 (7)	0.0230 (7)	0.0054 (5)	0.0025 (5)	0.0084 (6)
C4	0.0311 (7)	0.0146 (6)	0.0332 (8)	0.0021 (5)	0.0042 (6)	0.0075 (6)
C5	0.0244 (7)	0.0144 (6)	0.0318 (8)	−0.0016 (5)	−0.0009 (6)	−0.0009 (6)
C6	0.0222 (6)	0.0105 (6)	0.0162 (6)	−0.0025 (5)	−0.0060 (5)	0.0009 (5)
C7	0.0194 (6)	0.0181 (6)	0.0216 (7)	−0.0023 (5)	−0.0020 (5)	0.0006 (5)
C8	0.0253 (7)	0.0216 (7)	0.0182 (7)	−0.0050 (5)	0.0004 (5)	0.0020 (5)
C9	0.0272 (7)	0.0156 (6)	0.0156 (6)	−0.0040 (5)	−0.0055 (5)	0.0027 (5)
C10	0.0219 (6)	0.0126 (6)	0.0189 (7)	−0.0003 (5)	−0.0041 (5)	0.0004 (5)
C11	0.0233 (6)	0.0096 (6)	0.0151 (6)	−0.0019 (5)	−0.0018 (5)	0.0002 (5)
C12	0.0243 (7)	0.0282 (8)	0.0226 (7)	0.0043 (6)	0.0004 (5)	0.0000 (6)

### Geometric parameters (Å, °)

**Table:**

O1—C1	1.3518 (15)	C5—H5	0.9500
O1—C6	1.4075 (14)	C6—C7	1.3735 (18)
O2—C11	1.3630 (15)	C6—C11	1.3947 (18)
O2—C12	1.4329 (16)	C7—C8	1.3934 (18)
O3—N2	1.2211 (16)	C7—H7	0.9500
O4—N2	1.2295 (14)	C8—C9	1.3788 (19)
N1—C1	1.3217 (16)	C8—H8	0.9500
N1—C5	1.3367 (17)	C9—C10	1.3860 (19)
N2—C2	1.4604 (17)	C9—H9	0.9500
C1—C2	1.4031 (17)	C10—C11	1.3935 (17)
C2—C3	1.3871 (19)	C10—H10	0.9500
C3—C4	1.378 (2)	C12—H12A	0.9800
C3—H3	0.9500	C12—H12B	0.9800
C4—C5	1.383 (2)	C12—H12C	0.9800
C4—H4	0.9500		
			
C1—O1—C6	116.64 (9)	C11—C6—O1	118.84 (11)
C11—O2—C12	116.62 (10)	C6—C7—C8	119.22 (12)
C1—N1—C5	118.72 (12)	C6—C7—H7	120.4
O3—N2—O4	123.18 (12)	C8—C7—H7	120.4
O3—N2—C2	119.18 (11)	C9—C8—C7	119.61 (12)
O4—N2—C2	117.63 (12)	C9—C8—H8	120.2
N1—C1—O1	117.84 (11)	C7—C8—H8	120.2
N1—C1—C2	121.57 (12)	C8—C9—C10	121.14 (12)
O1—C1—C2	120.57 (11)	C8—C9—H9	119.4
C3—C2—C1	118.97 (12)	C10—C9—H9	119.4
C3—C2—N2	117.89 (11)	C9—C10—C11	119.70 (12)
C1—C2—N2	123.14 (11)	C9—C10—H10	120.1
C4—C3—C2	119.24 (13)	C11—C10—H10	120.1
C4—C3—H3	120.4	O2—C11—C10	125.24 (12)
C2—C3—H3	120.4	O2—C11—C6	116.28 (11)
C3—C4—C5	117.71 (13)	C10—C11—C6	118.47 (12)
C3—C4—H4	121.1	O2—C12—H12A	109.5
C5—C4—H4	121.1	O2—C12—H12B	109.5
N1—C5—C4	123.76 (13)	H12A—C12—H12B	109.5
N1—C5—H5	118.1	O2—C12—H12C	109.5
C4—C5—H5	118.1	H12A—C12—H12C	109.5
C7—C6—C11	121.84 (11)	H12B—C12—H12C	109.5
C7—C6—O1	119.13 (11)		
			
C5—N1—C1—O1	−177.26 (11)	C3—C4—C5—N1	−1.7 (2)
C5—N1—C1—C2	1.09 (19)	C1—O1—C6—C7	98.47 (13)
C6—O1—C1—N1	−1.12 (16)	C1—O1—C6—C11	−86.40 (14)
C6—O1—C1—C2	−179.48 (11)	C11—C6—C7—C8	−0.36 (19)
N1—C1—C2—C3	−1.92 (19)	O1—C6—C7—C8	174.62 (11)
O1—C1—C2—C3	176.38 (11)	C6—C7—C8—C9	−0.73 (19)
N1—C1—C2—N2	177.31 (11)	C7—C8—C9—C10	1.1 (2)
O1—C1—C2—N2	−4.40 (18)	C8—C9—C10—C11	−0.40 (19)
O3—N2—C2—C3	172.79 (12)	C12—O2—C11—C10	−1.44 (18)
O4—N2—C2—C3	−6.00 (17)	C12—O2—C11—C6	179.69 (11)
O3—N2—C2—C1	−6.45 (19)	C9—C10—C11—O2	−179.52 (11)
O4—N2—C2—C1	174.76 (12)	C9—C10—C11—C6	−0.68 (18)
C1—C2—C3—C4	0.92 (19)	C7—C6—C11—O2	−179.99 (11)
N2—C2—C3—C4	−178.35 (12)	O1—C6—C11—O2	5.02 (16)
C2—C3—C4—C5	0.8 (2)	C7—C6—C11—C10	1.06 (18)
C1—N1—C5—C4	0.8 (2)	O1—C6—C11—C10	−173.93 (11)

### Hydrogen-bond geometry (Å, °)

**Table:**

*D*—H···*A*	*D*—H	H···*A*	*D*···*A*	*D*—H···*A*
C3—H3···O2^i^	0.95	2.58	3.4085 (17)	146
C9—H9···O4^ii^	0.95	2.55	3.2659 (16)	132
C12—H12a···O3^iii^	0.98	2.52	3.3560 (18)	143

Symmetry codes: (i) −*x*+1, −*y*+1, −*z*; (ii) *x*+1/2, −*y*+1/2, *z*+1/2; (iii) *x*+1, *y*, *z*.
